# Virulence Specificity of the *Puccinia graminis* f. sp. *tritici* Population Originating from Durum Wheat in the Altai Region of Western Siberia, Russia

**DOI:** 10.3390/jof12060391

**Published:** 2026-05-29

**Authors:** Ekaterina S. Skolotneva, Vasiliy N. Kelbin, Margarita A. Rozova, Evsey Kosman

**Affiliations:** 1 Institute of Cytology and Genetics, Siberian Branch of Russian Academy of Sciences, Novosibirsk 630090, Russia; sk-ska@yandex.ru (E.S.S.);; 2 Federal State Budgetary Research Institution “Federal Altai Scientific Center for Agrobiotechnologies”, Barnaul 656910, Russia; mrosova@yandex.ru; 3 Institute for Cereal Crops Research, School of Plant Sciences and Food Security, Faculty of Life Sciences, Tel Aviv University, Ramat Aviv, Tel Aviv 6139001, Israel

**Keywords:** hard wheat, pathogen race, stem rust, *Triticum aestivum*, *Triticum durum*, virulence phenotype, effective number of different isolates, effective number of different populations, isolate singularity

## Abstract

For the first time, a race survey of *Puccinia graminis* f. sp. *tritici* (*Pgt*) population was conducted on *Triticum durum* in the Altai region of Western Siberia, Russia. A total of 34 single-pustule isolates with different virulence phenotypes were identified on durum wheat (*Triticum durum*) in 2025 and compared with *Pgt* from bread wheat (*Triticum aestivum*). The UPGMA-based clustering separated *Pgt* isolates into two distinct groups, suggesting the host-driven differentiation that was further proven using population genetics tools. The pathogen isolates from durum showed a wider range of virulence complexity, higher variability, and greater average singularity. Virulence frequencies of *Pgt* on *T. durum* and *T. aestivum* differed markedly for *Sr6*, *Sr7b*, *Sr9e*, *Sr17*+*13*, and several other genes, while *Sr24* and *Sr31* remained effective independently of the pathogen origin. Two races, PKCSF and NFMSF, were detected on both the hosts, indicating a shared pathogen gene pool between bread and durum wheat. Even assuming host-specific divergence of *Pgt* in the Altai region, there is a need for the deployment of the same resistance genes into both *T. aestivum* and *T. durum* cultivars to prevent an outbreak of stem rust in the event of favorable conditions for inoculum exchange between crops.

## 1. Introduction

Stem rust is an epidemic disease of wheat crops caused by the fungus *Puccinia graminis* f. sp. *tritici* (*Pgt*) [1]. While resistance (*Sr*) genes in wheat cultivars offer a means of controlling stem rust outbreaks, the pathogen evolves permanently, and newly emerged virulences can be hazardous to cultivated bread wheat (common wheat; *Triticum aestivum*) [2] and durum wheat (hard wheat; *Triticum durum*) [3].

Hexaploid *T. aestivum* is the second-most-consumed food crop globally [4,5] that provides the most widely sold grain [6]. Therefore, rusts on bread wheat (*P. graminis*, *P. triticina*, *P. striiformis*) have been better studied worldwide, both in terms of virulence markers and race dynamics. In particular, large-scale bread wheat surveillance networks have been coordinated for a long time with the Global Rust Reference Center and the USDA-ARS Cereal Disease Laboratory [7].

Tetraploid *T. durum* represents only about 5% of total wheat production [5]. Nevertheless, the importance of durum wheat in world agriculture is currently high due to a strategic advantage in an era of climate change. Unlike bread wheat, durum is better adapted to high temperatures and semi-arid climates, which makes it a vital resource for maintaining agricultural productivity in environments where many other crops struggle [8]. Durum wheat is the essential raw material for culturally significant food products such as pasta, couscous, bulgur, and certain traditional breads [9].

In modern production of durum wheat grain, the Russian Federation accounts for 4–6% of the global output [10] and is consistently listed among the top 10 largest producers of pasta products [11]. In general, the sown area of durum wheat varies significantly across years and periods. By the beginning of 2020, the area under durum wheat in Russia had decreased to 640 thousand hectares; the sown area increased and reached 1 million hectares in 2025, accounting for 6–9% of the total wheat area. Due to high yields (21.8 dt/ha for spring durum wheat and 34.2 dt/ha for winter durum wheat), production reached 2125 thousand tons in 2025 [4]. Approximately 80% of Russian production of the durum wheat grain is concentrated in the Orenburg and Chelyabinsk oblasts, as well as in the Volga and West Siberian regions [12]. The Altai region of Western Siberia is a traditional growing area for spring durum wheat. In 2025, durum wheat was cultivated on 58 thousand hectares in Altai, with the sown area distributed across zones providing favorable conditions: the steppe zone (70%), the forest–steppe zone (2%), and the dry-steppe foothills (28%).

Stem rust occurs on durum wheat crop in the Altai region relatively often; however, severe disease outbreaks occur rarely [13,14]. The disease usually develops in the second half of July, when early-maturing varieties are at the late milk to early dough stage. Therefore, early sowing dates are locally recommended to help plants escape the disease and thus to avoid epidemics. Based on observations during years of high disease pressure, highly susceptible varieties exhibited substantially lower yields compared to varieties with low disease incidence [13,14]. Despite being located in Western Siberia, Russia, where stem rust monitoring takes place regularly, Altai was rarely included in the surveys. No reports on *Pgt* on durum wheat and the population structure of the pathogen have been published for the past several decades. Thus, the main objective of our research was to study virulence variability and the structure of the *Pgt* population on durum wheat in the Altai region of Western Siberia.

## 2. Materials and Methods

### 2.1. Sampling Sites

*Pgt* sampling was conducted in 2025 from plants of durum wheat at the Federal Altai Scientific Center for AgroBiotechnologies (FASCA)—Nauchnyj gorodok (53°26′00″ N, 83°26′00″ E), located at an altitude of 197 m above sea level in the Ob’ forest–steppe zone on the bank of the Ob’ River. The study site is characterized by an annual precipitation of 422 mm, with 207 mm falling during the spring crop season (May–August). The mean minimum temperature in January is −16.4 °C, and the mean maximum temperature in July is 19.8 °C [15]. During the cropping season, relative humidity (RH) at this location ranges between 55% and 70%. The soils are classified as leached chernozems, low in humus, mid-deep, medium loamy, and suitable for crop production. During the field survey in 2025, the first detailed study of stem rust was conducted in the wheat breeding nursery. Early symptoms of stem rust appeared on durum wheat in the last ten days of July at the milk stage, whereas *Pgt* spores were sampled at the beginning of the wax stage at the end of August. In the period from 2017 to 2025, infected stems of bread wheat with uridiniospores of *Pgt* were obtained from local germplasm grown in commercial fields of the Altai region.

### 2.2. Wheat Varieties

*Pgt* sampling was carried out on three varieties of durum wheat: Oazis, ATP Prima, and ATP Prima/Durtres hybrid line. Varieties Oazis and ATP Prima are of local origin, whereas Durtres was derived from European germplasm of durum wheat [16]. All three varieties are tall, of late-ripening type, with released yield potential up to 7.5 t/ha. Oazis is susceptible to natural and artificial inoculation; it usually serves as a local check for stem rust, while ATP Prima is mid-susceptible to stem rust. In the conditions of the Altai region, Durtres is a semi-dwarf early-ripening variety that usually escapes stem rust development.

### 2.3. Samples of Pgt: Storage and Recovery

Bulk field samples were stored in an automatic desiccator under dry cold conditions until processing. Recovery of *Pgt* from durum and bread wheat samples and verification of the applicability of Koch’s Postulate were conducted using the susceptible cv. Oazis and line Khakasskaya, respectively, at the laboratory of the Institute of Cytology and Genetics of the Siberian Branch of the Russian Academy of Sciences (ICG SB RAS). A total of 34 and 49 single pustule isolates were obtained from durum and bread wheat samples, respectively, according to the protocol adopted for the isolation of wheat stem rust [2]. All *Pgt* isolates were used for virulence phenotyping and race identification by seedling tests.

### 2.4. Seedling Tests for Virulence and Race Identification

Virulence phenotypes were determined using a set of 20 North American wheat differential lines carrying the following *Sr* genes: *Sr5* (ISr5 Ra), *Sr21* (CnS_Triticum monoc. Deriv.), *Sr9e* (Vernstein), *Sr7b* (ISr7b Ra), *Sr11* (ISr11 Ra), Sr6 (ISr6a Ra), *Sr8a* (ISr8a Ra), *Sr9g* (CnSr9g), *Sr36* (W2691SrTt 1), *Sr9b* (W2691Sr9b), *Sr30* (BtSr30Wst), *Sr17*+*13* (Combination VII), *Sr9a* (ISr9a Ra), *Sr9d* (ISr9d Ra), *Sr10* (W2691Sr10), *SrTmp* (CnsSrTmp), *Sr24* (LcSr24Ag), *Sr31* (Benno Sr31/6∗LMPG), *Sr38* (VPM 1), and *SrMcN* (McNair 701). Susceptible checks (line Khakasskaya and cv. Oazis for isolates from bread and durum wheat, respectively) were included in each experiment.

The inoculation and assessment procedures followed the protocols adopted for wheat stem rust [2,17,18]. For each isolate, urediniospores were suspended in Novec 7100 oil (Electronics Markets Materials Division 3M Center, St. Paul, MN, USA) and applied with an airbrush to 5–10 seedlings per pot at the one-leaf stage. Inoculated plants were placed in a dark dew chamber at 18 °C ± 2 °C for 24 h and then transferred to a growth room maintained at 20 °C ± 2 °C with a 16 h photoperiod. To prevent cross-contamination, the entire set of inoculated plants was covered with a cellophane box.

Infection types (IT) were identified 14–16 days after inoculation using the modified Stakman scale [19]. IT scores from 0 to 2 were interpreted as low (avirulent), while IT scores 3 and 4 were considered high (virulent). Each single-pustule isolate was tested at least twice on the full set of differential lines.

Races were designated following the North American (hexadecimal) coding system for *Pgt* [17,18]. Based on the virulence patterns of isolates obtained with the standard set of 20 Sr differentials, the race of each isolate was determined and converted into the corresponding hexadecimal code.

### 2.5. Data Analysis

Analyses of variability within and among the annual *Pgt* collections of isolates from *T. durum* in 2025 and *T. aestivum* in 2017, 2018, and 2025 in the Altai region of Russia were performed separately for isolate and race data (pools of all *T. aestivum* isolates and races were also considered). Descriptive parameters, such as virulence frequency and relative virulence complexity RVC [20], were calculated for collections of interest. Statistical significance of distinction in virulence frequencies on each differential line in any two *Pgt* collections was estimated based on the 95% confidence interval of the binomial distribution for a difference between the corresponding virulence frequencies (means of the corresponding binomial distributions).

The simple mismatch dissimilarities between virulence profiles of isolates and races were calculated and employed to study the structure, relationships, and diversity of the *Pgt* collections using the assignment-based approach. The corresponding *KW* dispersion within and *KB* distance between collections were calculated [21,22,23]. Differentiation among collections was estimated with the permutation test (1000 random partitions) for differentiation statistics ${dif}_{KW}$ (Equation (1) in [24,25]) based on the *KW* dispersion (see also Equation (13) in [23,26]).

The number of effectively different isolates and races within a population DT,KW1 was estimated with the metric of functional trait dispersion (Equations (5) and (6) in [27] for M=KW, and [28]). The effective number ranges from 1 (all isolates/races are identical) to an actual number of isolates/races when they are absolutely different. The normalized version of this indicator nDT,KW1 (corrected Equation (5) in [29]; Equation (3) in [30]) expresses an extent of variability within a collection independently of sample size (actual number of isolates/races in a given collection).

Extraordinary isolates within a collection were revealed using the so-called metric of individual singularity (Equations (1)–(3) in [31]; Equations (1)–(5) in [32]). The singularity of each *Pgt* isolate was estimated based on the simple mismatch dissimilarity of that isolate from all other isolates in the collection in question. The singularity of a whole collection was calculated as the average singularity of all isolates that belong to it (Equations (7) and (8) in [31]).

The effective number of different *Pgt* collections DADWKB1 was calculated based on the *KB* distances between collections (only polymorphic virulence loci were included) and the $ADW={ADW}_{KB}$ dispersion (*Average Distance* between collections *Within* a given set of collections) with regard to the *KB* distance according to [33]. The effective number ranges from 1 (all collections are identical) to an actual number of collections when they are absolutely different.

Some of the above-mentioned metrics used in this study were developed recently, and they can be interpreted as follows. The effective number of different isolates/races quantifies how distinct and equidistant (evenly distributed) the virulence profiles of individual isolates/races are in the corresponding pool. The effective number of different collections quantifies how distinct and equidistant (evenly distributed) collections of isolates/races are in the corresponding pool. Individual singularity quantifies how different (distinct and equidistant from others) an individual isolate’s virulence profile is relative to all other isolates in the pool. All metrics are based on a proper measure of dissimilarity between virulence profiles or distance between collections. Since the values of the mentioned parameters depend on the sample size of units in question (number of isolates/races or collections in a given pool), the normalized versions of those indicators with values in $\left[0,\;1\right]$ interval are utilized to express the extent of variability or singularity independently of sample size. The normalized metrics are needed for a valid comparison of pools with different sample sizes.

UPGMA dendrogram of relationships among the races in all *Pgt* collections with regard to the simple mismatch dissimilarity between them was generated using the SAHN program of the NTSYSpc package, version 2.2 (Exeter Software, Setauket, NY, USA). Other above-mentioned calculations were performed with VIRULENCE ANALYSIS TOOL (VAT) software [34,35] (accessed on 25 April 2026) and FUNCTIONAL DIVERSITY ANALYSIS TOOLS (FDAT) software (accessed on 25 April 2026). Both packages are available at https://en-lifesci.tau.ac.il/profile/kosman (accessed on 25 April 2026).

## 3. Results

In the 2025 field survey, initial sporulating pustules of *Pgt* were observed on durum wheat in the last ten days of July at the milk stage. This survey included the first detailed nursery observation. About 30% of the late-ripening durum varieties were found rusted by the second part of August, and the studied collection of *Pgt* urediniospores was sampled at that time. Disease severity of *Pgt* on Oazis, ATP Prima, and ATP Prima/Durtres hybrid line was evaluated within the range from medium susceptibility to high susceptibility.

In total, 34 and 49 *Pgt* isolates originating from durum wheat in 2025 and bread wheat in 2017 (20 isolates), 2018 (14), and 2025 (15) in the Altai region of Russia were tested (Table 1). Altogether, eight different races originating from *T. aestivum* (seven in 2017, and four in both 2018 and 2025) and seven from *T. durum* were detected on the standard set of 20 differentials. Characterization of all races and their hexadecimal codes determined according to the North American encoding system of race designations is shown in Table 2.

### 3.1. Relationships Among the Pgt Races of Different Origin

**Figure 1:**
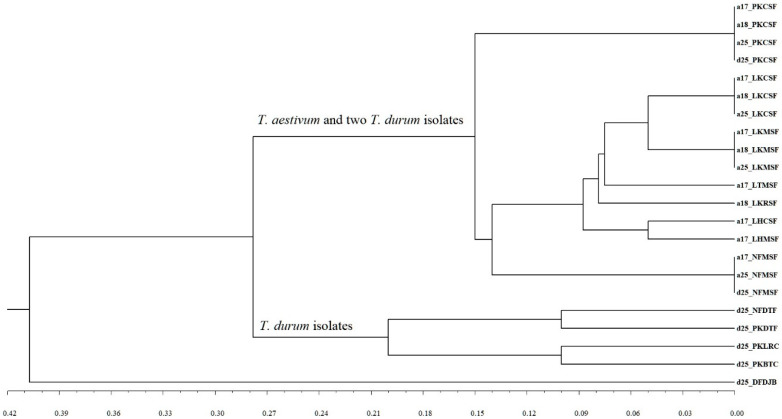
The UPGMA dendrogram of relationships among the *Puccinia graminis* f. sp. *tritici* isolates identified in the Altai region on durum (2025) and bread wheat (2017–2025). Each isolate code includes its origin, year of collection, and hexadecimal race designation: for example, a17_PKCSF means that this isolate originates from *T. aestivum* (a), was collected in 2017 (17), and belongs to the PKCSF race; d25_NFDTF means that this isolate originates from *T. durum* (d), was collected in 2025 (25), and belongs to the NFDTF race.

The UPGMA dendrogram generated with the simple mismatch dissimilarities between races (Figure 1) demonstrated a clear difference between *Pgt* races identified in the collection from *T. durum* versus the collections from *T. aestivum*, though two races (PKCSF and NFMSF) were common for both hosts (Table 2).

### 3.2. Virulence and Race Characterization

**Figure 2:**
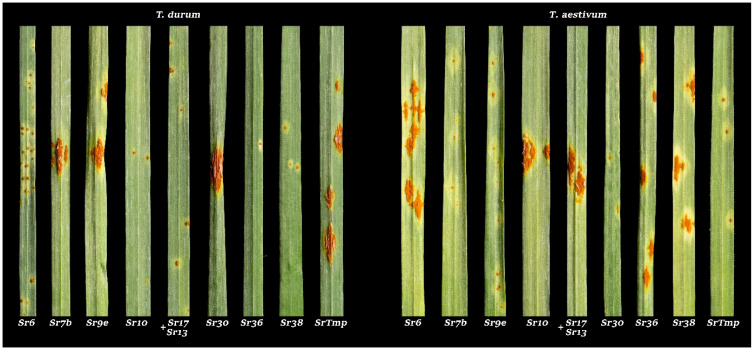
Infection types produced by the “durum” races (**left**) vs. the “bread” races (**right**) of *Puccinia graminis* f. sp. *tritici* on differential lines with *Sr6*, *Sr7b*, *Sr9e*, *Sr10*, *Sr17*+*13*, *Sr30*, *Sr36*, *Sr38*, and *SrTmp*.

Virulence to 15 *Sr* genes (among 20 tested) was detected at various frequencies (Table 3). No virulence to *Sr21*, *Sr24*, and *Sr31* was detected in all isolates tested, whereas *Sr9g* and *Sr9d* were ineffective against all isolates. Very high virulence frequencies (≥0.85) were observed for resistance genes *Sr5*, *Sr8a*, *Sr9a*, *Sr10*, and *SrMcN*. Significant variation in virulence frequency among the *Pgt* collections from *T. aestivum* and *T. durum* was observed for resistance genes *Sr9e*, *Sr7b*, *Sr6*, *Sr36*, *Sr30*, *Sr17*+*Sr13*, *Sr10*, *SrTmp*, and *Sr38*: virulence frequencies on *Sr9e*, *Sr7b*, *Sr30*, and *SrTmp* were considerably higher for *Pgt* from *T. durum*, whereas isolates from *T. aestivum* were characterized by higher virulence frequencies on *Sr6*, *Sr36*, *Sr17*+*Sr13*, *Sr10*, and *Sr38* (Table 3, Figure 2).

Two common races, PKCSF and NFMSF, for *T. durum* and *T. aestivum* hosts were the only two that were virulent to *Sr17*+*Sr13* among all seven “durum” races, whereas all “aestivum” races were virulent on that resistance gene. The average relative virulence complexity of *Pgt* isolates was similar (average RVC = 0.55; around 11 virulences of 20) for all collections. However, the RVC variance was much higher for the collection from *T. durum*, with the RVC range between 0.3 and 0.65 versus the range 0.45–0.6 for the collection from *T. aestivum* (Table 3). Thus, “durum” races DFDJB and PKDTF had the lowest (0.3) and highest (0.65) RVC values with 6 and 13 virulences of 20, respectively.

The *Pgt* isolates originating from *T. durum* were much more singular than those from *T. aestivum*, with the average normalized singularity nS = 0.165 vs. 0.071 (Table 3). A range of isolate singularities was wider for the “durum” isolates as well, 0.114–0.280 vs. 0.044–0.108, whereas the relative variation in the isolate singularities was nearly the same for both the collections [e.g., (0.280–0.114)/0.280 = 0.593 and (0.108–0.044)/0.108 = 0.592]. The most singular “durum” isolates (nS = 0.280) belonged to the race DFDJB with the lowest virulence complexity (RVC = 0.3).

### 3.3. Variability Within and Among Pgt Collections

Variability within the *Pgt* collection of isolates originating from *T. durum* was considerably higher as compared with the collections from *T. aestivum*, as follows from estimates of both the dispersion *KW* and the normalized effective number of different isolates *nENDI* (Table 1): KW=0.30 vs. KW=0.13÷0.15, and nENDI=0.39 vs. nENDI=0.16÷0.20). The simple richness estimate was larger for the “durum” collection only versus the pool of isolates originating from *T. aestivum* (0.21 vs. 0.16, respectively).

No significant pairwise differentiation was revealed among the *Pgt* collections of isolates from *T. aestivum* in 2017, 2018, and 2025 (Table 4), and distances between them were very small (KB=0.014÷0.043). The effective number of different *T. aestivum* collections equals 1.08 of the three original ones; this means that the three annual *Pgt* collections of isolates from *T. aestivum* were practically indistinguishable. On the other hand, the *Pgt* collection of isolates originating from *T. durum* in 2025 was significantly different (p=0.01) and rather distant (KB=0.220÷0.248) from each of the annual “aestivum” collections (Table 4) as well as from the collection of all *T. aestivum* isolates.

## 4. Discussion

In the Altai region, stem rust was sporadically recorded on bread wheat crops from the heading stage to the end of grain maturation. The overall disease severity usually ranged from medium resistance to medium susceptibility.

The 2025 growing season in the durum wheat production area within the Altai region was characterized by an unusually prolonged vegetative period and meteorological conditions highly favorable to the development of stem rust. The month of August was notably cool and rainy, and abundant precipitation resulted in elevated humidity levels: daytime temperatures ranged from 20 to 25 °C, with brief warmer periods, and prolonged periods of drought were lacking.

These specific weather conditions may influence two important agronomic and epidemiological factors that likely contributed to the stem rust outbreak: (i) host phenology and delayed senescence, and (ii) pathogen proliferation and re-infection cycles. The moderate temperatures and consistent moisture availability may have resulted in a slowing maturation rhythm for spring wheat accessions in the field nursery; plants could remain physiologically active and susceptible to infection through the end of August. As determined for stem rust [36], the combination of high humidity and sustained temperatures within the 20–25 °C range provided an optimal environment for *Pgt* urediniospore germination, infection, and subsequent sporulation, a shortened latent period, and an increasing number of successive cycles of infection on a susceptible host. The mentioned conditions were observed in the Altai region from the end of July to the end of August 2025; thus, they might stimulate the emergence and stability of stem rust on durum wheat.

Screening for resistance of durum wheat germplasm in the Russian producing areas (mainly, the Volga region and Western Siberia, including the Altai region) has highlighted an acute need for monitoring of *Pgt* on both wheat crops, *T. aestivum* and *T. durum* [13,14]. However, large-scale studies of *Pgt* on durum are still lacking in Russia. In 2025, a survey of wheat stem rust first included durum germplasm in Altai.

Two common *Pgt* races (PKCSF and NFMSF) detected in both *T. aestivum* and *T. durum* hosts are evidence of a shared gene pool of the pathogen on wheat crops in the Altai region. This finding corresponds to global monitoring data on *Pgt* from bread and durum wheats obtained during the last decades: some races do not differentiate between *T. aestivum* and *T. durum* or may move from one host to another. For example, the so-called “Sicily race” TTRTF was identified on bread wheat and durum in Israel [37], Italy, Spain, Tunisia, and Iran [38]; it was also tracked across other Mediterranean regions and Central Europe [2].

There were two clearly separated clusters of *Pgt* isolates in the UPGMA dendrogram (Figure 1): one with isolates from *T. durum* and another one with isolates mostly sampled from *T. aestivum*. Therefore, despite the existence of shared races, the *Pgt* isolates originating from bread wheat and durum wheat were mostly distinct, which supports a hypothesis of host-driven differentiation. A similar trend of commonality and uniqueness of pathogen isolates originated from bread wheat and durum was established for *Puccinia striiformis* (*Pst*) in the European part of Russia: only a few *Pst* races were shared by the two hosts [39]. Global studies of *Puccinia triticina* population on durum versus bread wheat also declare genetic isolation between samples by virulence and SSR markers [14,26,40].

Based on the analogy of pathogen differentiation among populations from durum and bread wheat for the most important fungal pathogens of wheat, hypotheses of independent evolution and/or adaptation to specific regional conditions seem quite probable for the *Pgt* population on durum in Altai. This premise can be indirectly supported by differences in the key indicators: relative virulence complexity (RVC), variability, and singularity. First, RVC values were of a wider range for the “durum” *Pgt* isolates (0.3–0.65 vs. 0.45–0.6 for the “bread” isolates). Unlike the virulence patterns of isolates from *T. aestivum* with a moderate number of virulence alleles, the patterns of isolates from *T. durum* included two extremes: race DFDJB with only six out of 20 possible virulences (RVC = 0.3; Table 3) and race PKDTF with 13 virulences (RVC = 0.65). Second, the *Pgt* isolates on durum were of much higher variability (*nENDI* = 0.39 vs. 0.19; Table 1) and average singularity (nS = 0.165 vs. 0.071; Table 3). Thus, the *Pgt* population on durum wheat seems to be of high virulence variability that may ensure a pathogen plasticity: rapid response to environmental changes, viability, and survival within a growing season. This specific need in keeping highly diverse *Pgt* isolates on durum wheat and the tendency towards such microevolution could reflect the generally unfavorable epidemiological conditions for persistent stem rust infection of *T. durum* in the Altai region.

Variability of the *Pgt* population originating from bread wheat was very stable over time, with no signs of differentiation among the annual *Pgt* collections (Table 1, Table 3 and Table 4). This indicates that the *Pgt* population from *T. aestivum* in the Altai region is shaped by stable selection forces, so that the same well-adapted pathogen virulence phenotypes and genotypes are maintained in the population from year to year. However, note that each of the annual pathogen collections from bread wheat differed significantly from the *Pgt* collected in Altai from durum in 2025 (Table 4).

Virulence frequencies of *Pgt* isolates from durum and bread wheat were quite different, especially on the differential lines with *Sr9e*, *Sr7b*, *Sr6*, *Sr36*, *Sr30*, *Sr17*+*13*, *Sr10*, *SrTmp*, and *Sr38* resistance genes (Table 3). Only *Sr21*, *Sr24*, and *Sr31* genes were equally effective across *Pgt* populations on bread and durum wheat in Altai. Note that *Sr21* is ineffective in most areas of Western Siberia except the Kemerovo region [41], but *Sr24* and *Sr31* remain effective; thus, they are commonly deployed genes in wheat breeding against stem rust in Russia [42].

As mentioned before, two races, PKCSF and NFMSF, with virulence to *Sr9e* and *Sr17*+*13*, were isolated from both the crops in the Altai region. The use of resistance genes *Sr9e* and *Sr13* has long been of particular importance in the breeding of *T. durum* germplasm in North America and worldwide because of their effectiveness against all known variants of the Ug99 race group [43]. Since the first detection of the JRCQC and TRTTF races in Ethiopia, the virulence combination of *Sr9e* and *Sr13* has been reported in North Africa and the Middle East, where durum wheat is traditionally grown [3]. However, it is reasonable to assume that the combined virulence to *Sr9e* and *Sr13* genes in the Altai region arose independently of the global spread of the “Ethiopian” races because Altai is an extremely remote area from North Africa and the Middle East. However, it is important to consider an alternative explanation for the presence of these virulent races on durum wheat in the Altai region. There were highly abundant races PKCSF and NFMSF in the local *T. aestivum* pool (accounting for 12 and seven isolates, respectively, Table 2). This raises the possibility that the “shared races” detected on durum wheat may result from direct spore spillover from neighboring commercial bread wheat fields during peak spore-release cycles, rather than representing an independent microevolutionary event. In any case, the coincidence of the same damaging combination of virulences in very distant world regions seems to be an interesting issue for further study aimed, in particular, to clarify the relative contribution of migration versus de novo adaptation in shaping regional pathogen populations.

Summarizing outcomes obtained with all measurable indicators, one can point to highly dynamic processes shaping the *Pgt* population from durum; this also raises a hypothesis of divergence of *Pgt* on *T. aestivum* and *T. durum* in the Altai region. While the former is stable and rather predictable, the latter appears to be variable and has the potential to change rapidly along with the local (or regional) shifts in conditions. However, it is critical to acknowledge a key limitation: historical baseline data for stem rust on durum wheat in this region have been lacking. Until the surveys are accumulated in the region, it remains unclear whether the high diversity of “durum” *Pgt* reflects the specificity of the local population or is caused by temporary outbreaks. Further validation, including SSR analysis, is required. Even assuming host-specific divergence of *Pgt* in the Altai region, there is a need for the deployment of the same resistance genes into both *T. aestivum* and *T. durum* cultivars to prevent an outbreak of stem rust in the event of favorable conditions for inoculum exchange between crops.

## Figures and Tables

**Table 1:** Variability within the *Pgt* collections from *T. aestivum* (*Tae*) and *T. durum* (*Tdu*).

	Number (*N*) of Isolates	Number (*NR*) of Races ^a^	Simple Richness ^b^	Dispersion KW	ENDI ^c^	nENDI ^d^
*Tae* 2017	20	7	0.35	0.14	2.10	0.18
*Tae* 2018	14	4	0.29	0.13	1.48	0.16
*Tae* 2025	15	4	0.27	0.15	1.59	0.20
*Tdu* 2025	34	7	0.21	0.30	3.31	0.39
*Tae* pool	49	8	0.16	0.14	2.24	0.18

^a^ number of races based on the standard set of 20 differentials; ^b^ number of races relative to the corresponding number of isolates: NR/N; ^c^ effective number of different isolates ENDI=DT,KW1 (based on 15 polymorphic virulence loci); ^d^ normalized effective number of different isolates: nENDI=DT,KW1−1/N−1.

**Table 2:** Characterization of *Pgt* races originating from *T. aestivum* (*Tae*) and *T. durum* (*Tdu*) in the Altai region, Russia.

No ^a^	a/v Formula ^b^	RVC ^c^	Race ^d^	Origin ^e^	Abundance ^f^
1	*7b*, *8a*, *9b*, *9e*, *11*, *30*, *36*, *Tmp/**5*****, *6*, *9a*, *10*, *17*+*13*, *38*, *McN***	0.45	LHCSF	*Tae* 2017	1
2	*7b*, *8a*, *9b*, *9e*, *11*, *30*, *Tmp/**5*****, *6*, *9a*, *10*, *17*+*13*, *36*, *38*, *McN***	0.5	LHMSF	*Tae* 2017	2
3	*7b*, *9b*, *9e*, *11*, *30*, *36*, *Tmp/**5*****, *6*, *8a*, *9a*, *10*, *17*+*13*, *38*, *McN***	0.5	LKCSF	*Tae* 2017, 18, 25	9
4	*7b*, *9b*, *9e*, *11*, *30*, *Tmp/**5*****, *6*, *8a*, *9a*, *10*, *17*+*13*, *36*, *38*, *McN***	0.55	LKMSF	*Tae* 2017, 18, 25	15
5	*7b*, *9e*, *11*, *30*, *Tmp/**5*****, *6*, *8a*, *9a*, *9b*, *10*, *17*+*13*, *36*, *38*, *McN***	0.6	LKRSF	*Tae* 2018	2
6	*7b*, *9b*, *9e*, *30*, *Tmp/**5*****, *6*, *8a*, *9a*, *10*, *11*, *17*+*13*, *36*, *38*, *McN***	0.6	LTMSF	*Tae* 2017	1
7	*5*, *6*, *7b*, *9a*, *9b*, *11*, *17*+*13*, *36*, *38*, *Tmp*, *McN/**8a*****, *9e*, *10*, *30***	0.3	DFDJB	*Tdu* 2025	4
8	*6*, *7b*, *9b*, *11*, *17*+*13*, *36/**5*****, *8a*, *9a*, *9e*, *10*, *30*, *38*, *Tmp*, *McN***	0.55	NFDTF	*Tdu* 2025	8
9	*9b*, *11*, *17*+*13*, *30*, *36*, *38/**5*****, *6*, *7b*, *8a*, *9a*, *9e*, *10*, *Tmp*, *McN***	0.55	PKBTC	*Tdu* 2025	5
10	*9b*, *11*, *17*+*13*, *36/**5*****, *6*, *7b*, *8a*, *9a*, *9e*, *10*, *30*, *38*, *Tmp*, *McN***	0.65	PKDTF	*Tdu* 2025	6
11	*9b*, *10*, *11*, *17*+*13*, *30*, *36*, *38/**5*****, *6*, *7b*, *8a*, *9a*, *9e*, *Tmp*, *McN***	0.55	PKLRC	*Tdu* 2025	5
12	*6*, *7b*, *9b*, *11*, *30*, *Tmp/**5*****, *8a*, *9a*, *9e*, *10*, *17*+*13*, *36*, *38*, *McN***	0.55	NFMSF	*Tae* 2017, 25	7
				*Tdu* 2025	3
13	*9b*, *11*, *30*, *36*, *Tmp/**5*****, *6*, *7b*, *8a*, *9a*, *9e*, *10*, *17*+*13*, *38*, *McN***	0.6	PKCSF	*Tae* 2017, 18, 25	12
				*Tdu* 2025	3

^a^ Race number; ^b^ Reduced a/v formula with virulences shown in **bold**; all isolates were avirulent to *Sr21*, *24*, *31* and virulent to *Sr9g*, *9d*; ^c^ Relative virulence complexity of the corresponding race; ^d^ Hexadecimal code of a/v formula (race); the encoding was based on the standard ordered set of 20 North American differentials; ^e^ Race origin; for example, *Tae* 2017, 18, 25 (*Tdu* 2025) means that the corresponding race was identified among isolates originating from *T. aestivum* (*T. durum*) in 2017, 2018 and 2025 (2025); ^f^ Number of isolates of a given race with the corresponding origin (in the pool of all three annual collections of *Pgt* isolates from *T. aestivum* and in the collection from *T. durum* in 2025).

**Table 3:** Virulence frequency, relative virulence complexity (RVC), and singularity (nS) of *Pgt* isolates in collections from *T. aestivum* (*Tae*) and *T. durum* (*Tdu*).

*Sr* Genes	*Tae* 2017	*Tae* 2018	*Tae* 2025	*Tdu* 2025	*Tae* Pool
*Sr5*	1	1	1	0.88	1
*Sr21*	0	0	0	0	0
***Sr9e*** ^a^	0.45	0.21	0.47	1	0.39
***Sr7b***	0.25	0.21	0.27	0.56	0.24
*Sr11*	0.05	0	0	0	0.02
***Sr6***	0.8	1	0.8	0.56	0.86
*Sr8a*	0.85	1	1	1	0.94
*Sr9g*	1	1	1	1	1
***Sr36***	0.55	0.5	0.6	0.24	0.55
*Sr9b*	0	0.14	0	0	0.04
***Sr30***	0	0	0	0.53	0
***Sr17+13***	1	1	1	0.18	1
*Sr9a*	1	1	1	0.88	1
*Sr9d*	1	1	1	1	1
***Sr10***	1	1	1	0.85	1
***SrTmp***	0	0	0	0.71	0
*Sr24*	0	0	0	0	0
*Sr31*	0	0	0	0	0
***Sr38***	1	1	1	0.59	1
*SrMcN*	1	1	1	0.88	1
RVC ^b^	0.55	0.55	0.56	0.54	0.55
RVC range ^c^	0.45–0.6	0.5–0.6	0.5–0.6	0.3–0.65	0.45–0.6
singularity nS ^d^				0.165	0.071
nS range ^e^				0.114–0.280	0.044–0.108

^a^ *Sr*-genes with statistically significant differences in virulence frequencies between collections from *T. aestivum* and *T. durum* are marked in bold (*p*-value equals 0.05); ^b^ average relative virulence complexity (RVC) of all isolates in a given collection based on the standard set of 20 differentials; ^c^ RVC range of all isolates in a given collection based on the standard set of 20 differentials; ^d^ average normalized singularity (nS) of all isolates in a given collection. ^e^ range of normalized singularities of all isolates in a given collection.

**Table 4:** *KB* distance (above diagonal) and significance of pairwise differentiation ${dif}_{KW}$ (below diagonal) between *Pgt* collections from *T. aestivum* (*Tae*) and *T. durum* (*Tdu*).

	*Tae* 2017	*Tae* 2018	*Tae* 2025	*Tdu* 2025
*Tae* 2017 ^a^	0	0.043	0.014	0.229
*Tae* 2018	no ^b^	0	0.037	0.248
*Tae* 2025	no	no	0	0.220
*Tdu* 2025 ^a^	yes	yes	yes	0

^a^ *Tae* 2017 and *Tdu* 2025 mean *Pgt* collections originating from *T. aestivum* in 2017 and *T. durum* in 2025, respectively; ^b^ statistical significance of the pairwise differentiation; designation “yes” means rejection of the hypothesis of “no differentiation” at *p* = 0.01 level, whereas “no” means acceptance of that hypothesis at *p* = 0.05.

## Data Availability

The original contributions presented in this study are included in the article. Further inquiries can be directed to the corresponding author.
